# Dysfunctional Autophagy and Endolysosomal System in Neurodegenerative Diseases: Relevance and Therapeutic Options

**DOI:** 10.3389/fncel.2020.602116

**Published:** 2020-12-17

**Authors:** Silvia Giovedì, Margherita Maria Ravanelli, Barbara Parisi, Barbara Bettegazzi, Fabrizia Claudia Guarnieri

**Affiliations:** ^1^Department of Experimental Medicine, University of Genoa, Genoa, Italy; ^2^IRCCS, Ospedale Policlinico San Martino, Genoa, Italy; ^3^Vita-Salute San Raffaele University, Milan, Italy; ^4^Division of Neuroscience, Neuropsychopharmacology Unit, IRCCS San Raffaele Scientific Institute, Milan, Italy; ^5^Division of Neuroscience, Gene Therapy of Neurodegenerative Diseases Unit, IRCCS San Raffaele Scientific Institute, Milan, Italy; ^6^Institute of Neuroscience, National Research Council (CNR), Milan, Italy

**Keywords:** autophagy, endocytosis, lysosomes, neurodegenerative diseases, synapse

## Abstract

Autophagy and endolysosomal trafficking are crucial in neuronal development, function and survival. These processes ensure efficient removal of misfolded aggregation-prone proteins and damaged organelles, such as dysfunctional mitochondria, thus allowing the maintenance of proper cellular homeostasis. Beside this, emerging evidence has pointed to their involvement in the regulation of the synaptic proteome needed to guarantee an efficient neurotransmitter release and synaptic plasticity. Along this line, an intimate interplay between the molecular machinery regulating synaptic vesicle endocytosis and synaptic autophagy is emerging, suggesting that synaptic quality control mechanisms need to be tightly coupled to neurosecretion to secure release accuracy. Defects in autophagy and endolysosomal pathway have been associated with neuronal dysfunction and extensively reported in Alzheimer’s, Parkinson’s, Huntington’s and amyotrophic lateral sclerosis among other neurodegenerative diseases, with common features and emerging genetic bases. In this review, we focus on the multiple roles of autophagy and endolysosomal system in neuronal homeostasis and highlight how their defects probably contribute to synaptic default and neurodegeneration in the above-mentioned diseases, discussing the most recent options explored for therapeutic interventions.

## Introduction

**FIGURE 1:**
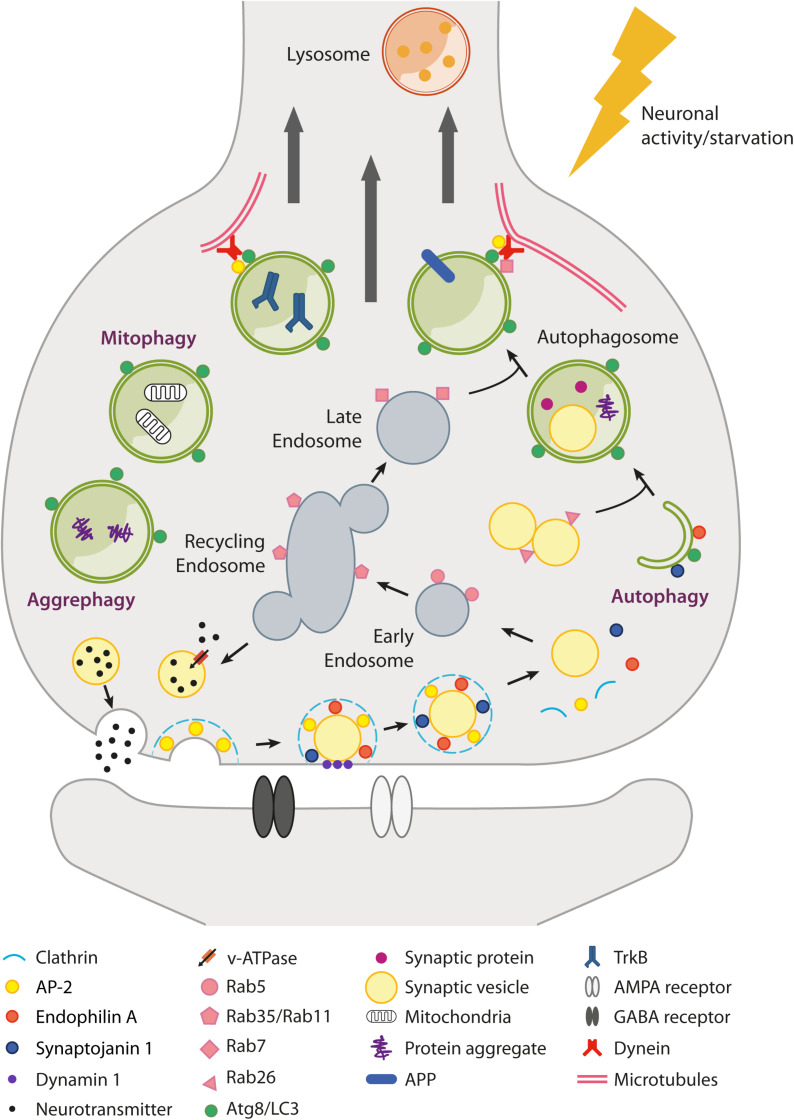
Schematic overview of autophagy and endolysosomal trafficking at the synapse. Local regulation of protein turnover occurs via autophagy (either non-selective and selective, e.g., mitophagy and aggrephagy) and the endolysosomal system (Rab5/Rab35/Rab7-dependent pathway), which can be stimulated by neuronal activity and nutrient depletion. In particular, metabolic signals activated upon nutrient restriction lead to mTORC1 inhibition and robust autophagy activation in many tissues. However, conflicting results have been raised as to whether starvation or mTORC1 inhibition are able to efficiently induce autophagy in neurons. mTORC1-independent pathways also exist (e.g., trehalose-induced) but are poorly characterized. At the presynaptic terminal, synaptic vesicle (SV) components retrieved after neurotransmitter release (e.g., by clathrin-mediated endocytosis) can transit through the endosomal compartment to be recycled back as newly formed SVs or be directed via late endosomes to lysosomes for degradation. Alternatively, SVs can be degraded via autophagy with a Rab26-dependent mechanism. Several presynaptic endocytic factors (e.g., Endophilin A, Synaptojanin1, AP-2) are implicated in the regulation of synaptic autophagy. Autophagosomes fuse with late endosomes to generate amphisomes and are transported along microtubules back to the soma, where fusion with lysosomes allows the digestion and recycling of cargoes. Autophagosomes can also act as signaling organelles, e.g., modulating neurotrophin-mediated TrkB signaling. Postsynaptically, autophagy and the endolysosomal system regulate neurotransmitter receptor trafficking and degradation.

Neurons are complex polarized post-mitotic cells, highly dependent on intracellular trafficking and protein quality control mechanisms to maintain proper homeostasis and sustain their function, particularly at the synaptic level (Wang et al., 2017; Wertz et al., 2020). One of the primary cellular degradative pathways is macroautophagy (hereafter autophagy), an evolutionarily conserved catabolic process that eliminates damaged organelles and misfolded proteins through the selective or non-selective engulfment of cytoplasmic materials in double-membrane autophagosomes. In neurons, autophagosome biogenesis is highly compartmentalized and extensively initiated in distal axons and at synapses in order to meet the need of constantly rejuvenating peripherally located proteins and organelles (Figure 1; Maday et al., 2012; Maday and Holzbaur, 2014). Autophagy intersects at multiple levels with the endolysosomal system (Winckler et al., 2018). Indeed, autophagosomes generated at synaptic terminals fuse with late endosomes, thus acquiring dynein motors and retrograde motility along microtubules (Gutierrez et al., 2004; Deinhardt et al., 2006; Cheng et al., 2015). During their retrograde journey, autophagosomes acidify and degrade their content by fusing with lysosomes, which are enriched at the soma (Lee et al., 2011; Maday et al., 2012). Autophagy induction is under the opposing control of the AMP-activated protein kinase (AMPK) and of the mammalian target of rapamycin complex 1 (mTORC1), which differentially phosphorylate the ULK1 autophagic initiating complex, leading to its activation or inhibition, respectively. Subsequently, a complex network of proteins regulates the biogenesis, maturation, and trafficking of neuronal autophagosomes (Bingol, 2018).

Autophagy inhibition, e.g., through genetic deletion of the autophagy-related genes 5 and 7 (ATG5, ATG7) involved in autophagosome biogenesis, leads to neurodegeneration in mice (Hara et al., 2006; Komatsu et al., 2006), thus supporting a fundamental role of this process in neuronal physiology and survival. In addition, emerging evidence has pointed to a role of autophagy and endolysosomal system in the turnover of synaptic components, such as synaptic vesicles (SVs), postsynaptic neurotransmitter receptors and mitochondria, thus opening the possibility that these pathways are involved in the shaping of synaptic structure and function (Liang, 2019). It is therefore not surprising that defects in autophagic and endolysosomal pathways have been implicated in the pathogenesis of several human neurodegenerative disorders such as Alzheimer’s disease (AD), Parkinson’s disease (PD), Huntington’s disease (HD) and amyotrophic lateral sclerosis (ALS), probably contributing to synaptic dysfunction and neurodegeneration (Winckler et al., 2018).

## Autophagy in Neuronal Homeostasis

### Autophagy at the Synapse

During development, autophagy promotes the assembly of presynaptic terminals at the neuromuscular junction in *Drosophila* (Shen and Ganetzky, 2009) and in specific neuronal subsets in *Caenorhabditis elegans* (Stavoe et al., 2016). In mouse hippocampal and cortical neurons, autophagy is required for developmental pruning of dendritic spines (Tang et al., 2014).

In mature synapses, the endolysosomal system and endosomal microautophagy (i.e., the engulfment of cargoes through the invagination of late endosome membranes generating multivesicular bodies) are important mechanisms for cytosolic and membrane-associated presynaptic protein sorting and turnover, particularly for SV components (Uytterhoeven et al., 2011, 2015; Fernandes et al., 2014; Sheehan et al., 2016; Jin et al., 2018). Synaptic autophagy is implicated as well in the degradation of specific SV proteins (Hoffmann et al., 2019) or of SVs via a Rab26-dependent pathway (Binotti et al., 2015). It is worth noting that the genetic ablation of the Rab26 guanine-nucleotide exchange factor Plekhg5 results in a late-onset motoneuron disease characterized by impaired autophagy, accumulation of aberrant SVs and degeneration of motoneuron axonal terminals (Lüningschrör et al., 2017). Interestingly, emerging evidence indicates that many presynaptic proteins involved in SV endocytosis play a role in synaptic autophagy: LRRK2, Endophilin-A and synaptojanin 1 are required for autophagosome formation (Murdoch et al., 2016; Soukup et al., 2016; Vanhauwaert et al., 2017), the adaptor proteins AP-2 and PICALM may act as cargo receptors (e.g., for the amyloid precursor protein APP; Tian et al., 2013), and AP-2 participates to autophagosome retrograde transport (Kononenko et al., 2017; Bera et al., 2020). In addition, the presynaptic scaffolding protein Bassoon inhibits autophagy by interacting with ATG5, and its loss leads to increased SV degradation (Okerlund et al., 2017). This molecular crosstalk between SV recycling and autophagy may provide an activity-dependent quality control mechanism needed to guarantee an adequate neurotransmitter release. Indeed, an efficient rejuvenation of SV proteins has been shown to facilitate neurotransmission in *Drosophila* (Uytterhoeven et al., 2011; Fernandes et al., 2014). On the other hand, in mouse dopaminergic neurons, the pharmacological induction of autophagy with the mTOR inhibitor rapamycin reduced the number of SVs and depressed the evoked dopamine release (Hernandez et al., 2012).

Concerning the postsynaptic compartment, the endolysosomal pathway has a well-known relevance in AMPA receptor recycling and degradation according to synaptic activity (Ehlers, 2000; Fernández-Monreal et al., 2012; Zheng et al., 2015). Autophagy is also emerging as being involved in the turnover of specific postsynaptic receptors, such as GABA_*A*_ and AMPA, thus participating to the fine-tuning of synaptic strength and plasticity (Rowland et al., 2006; Shehata et al., 2012, 2018; Hui et al., 2019; Shen et al., 2020). Scaffolding postsynaptic proteins such as PSD95, PICK1, and SHANK3 are substrates of autophagic degradation as well, implying that a modulation of autophagy can impact on the structural remodeling of dendritic spines by acting on these targets (Nikoletopoulou et al., 2017).

Given that the endolysosomal and autophagic pathways are strictly interconnected, whether they cooperate or are independently regulated for the sorting and degradation of specific cargoes, both at the presynaptic and postsynaptic side, is a matter of investigation. An interesting aspect is that synaptic autophagy is stimulated in response to neuronal activity (Shehata et al., 2012; Wang et al., 2015; Soukup et al., 2016; Hill et al., 2019), and may thus intervene under specific conditions. Along this line, recent evidence demonstrated that autophagy induction is required for activity-dependent structural and functional plasticity underlying novel memory formation *in vivo* (Glatigny et al., 2019). Lysosomes are also trafficked in an activity-dependent manner and can be recruited to dendritic spines upon local synaptic activation (Goo et al., 2017).

Further complicating the picture, besides their conventional function as degradative organelles, a subset of autophagosomes appears to act as signaling organelles able to retrogradely transport BDNF-activated TrkB receptors internalized at the presynapse toward the soma, thus unveiling a novel non-degradative role of autophagosomes at presynaptic boutons (Kononenko et al., 2017; Andres-Alonso et al., 2019).

### Mitophagy

Mitochondria provide energy and proper calcium buffering essential for synaptic activities, especially SV recycling (Rangaraju et al., 2014), thus the maintenance of an healthy mitochondrial network is fundamental for neuronal homeostasis. Mitophagy is the selective degradation of damaged or excess mitochondria by autophagy, but mitochondrial fragments may be engulfed by autophagosomes also in a constitutive non-selective process (Maday et al., 2012). Mitophagy can occur in both cell body and distal axons (Cai et al., 2012; Ashrafi et al., 2014; Evans and Holzbaur, 2020). Important mediators of the process, at least under stress conditions, are the Parkinson’s-related proteins PINK1 (PTEN-induced kinase 1) and the E3 ubiquitin ligase Parkin, which target defective mitochondria for ubiquitination and thus promote the recruitment of ubiquitin-binding autophagy receptors, such as p62/SQSTM1 and optineurin, and of the autophagic machinery (Matsuda et al., 2010; Wang et al., 2011). However, the evidence that loss of PINK1 or Parkin had no effect on basal mitophagy *in vivo* (Lee et al., 2018; McWilliams et al., 2018) suggested the existence of other PINK1/Parkin-independent mechanisms that cooperate in the clearance of dysfunctional mitochondria. Indeed, additional E3 ubiquitin ligases have been identified, as well as mitochondrial autophagic receptors, such as BNIP3, FUND1, and Bcl-2-L-13, which are expressed on the outer mitochondrial membrane and directly interact with LC3 to recruit the nascent phagophore on mitochondria (Villa et al., 2018). Along this line, an unconventional mitophagy receptor in neurons is the phospholipid cardiolipin: normally inserted in the inner mitochondrial membrane, it can be exposed on the mitochondrial surface upon mitochondrial depolarization and directly interacts with LC3 to recruit the autophagic machinery (Chu et al., 2013). Mitochondrial dysfunction and defective mitophagy have been described in various neurodegenerative diseases, including ALS, AD, and HD (Martinez-Vicente, 2017).

### Aggrephagy

Since neurons are non-dividing cells, they are particularly vulnerable to the accumulation of damaged or misfolded proteins. The degradation of misfolded protein aggregates is achieved through a selective process called aggrephagy, but some of this clearance is, however, non-selective (Maday et al., 2012; Wong and Holzbaur, 2014a). Several autophagy receptors, able to bind both the degradative targets and the forming autophagosome, have been implicated in aggrephagy (e.g., p62/SQSTM1, optineurin, NBR1, and Ubiquilin-2) (Deng et al., 2017). The binding affinity of autophagy receptors to specific cargoes and to core members of the autophagy machinery is modulated by post-translational modifications of the receptors and by the interaction with autophagy adaptors, such as Huntingtin (Htt) and Alfy/WDFY3. This regulates the efficiency and specificity of aggregate clearance, in ubiquitin-dependent and -independent manners (Deng et al., 2017; Turco et al., 2019). The accumulation in neurons of aggregated pathogenic proteins such as α-synuclein, tau, Aβ, and mutant Huntingtin (mHtt) is a common feature of several neurodegenerative disorders and is related to dysfunctional selective autophagy. Accordingly, genetic mutations in some autophagy receptors have been associated with ALS and frontotemporal lobar degeneration (Deng et al., 2017). In this context, the modulation of specific autophagy receptors may represent a valuable strategy to improve the proteotoxic burden in these disorders.

## Autophagy and Neurodegenerative Disorders

### Alzheimer’s Disease

Alzheimer’s disease is the most common form of neurodegenerative dementia, characterized by the extracellular accumulation of amyloid plaques, composed primarily of Amyloid-β (Aβ), and intracellular tau tangles. Defective autophagy and endolysosomal function have been extensively documented as early events in AD, and variants in several genes involved in endosomal trafficking (*SORL1, PICALM, CD2AP, BIN1, PLD3*) have been recognized as genetic risk factors (Guimas Almeida et al., 2018). Autophagic vacuoles (AVs) and enlarged endosomes accumulate in the brain of AD patients and mouse models, especially in dystrophic neurites and synaptic terminals (Cataldo et al., 2000; Nixon et al., 2005; Yu et al., 2005), likely as a result of impaired AV retrograde transport, maturation and lysosomal clearance (Boland et al., 2008; Lee et al., 2010; Tammineni et al., 2017).

In PS1/APP mice, dystrophic neurites filled with AVs are associated with Aβ plaques (Sanchez-Varo et al., 2012). Indeed, the autophagic and endolysosomal networks have been recognized as crucial sites for APP processing by β- and γ-secretases and for Aβ generation and secretion (Yu et al., 2005; Nilsson et al., 2013; Whyte et al., 2017). The regulated trafficking of APP and secretases through the autophagic and endolysosomal pathways probably determines the net balance between Aβ production and APP/Aβ lysosomal degradation. The levels of the autophagy initiation factor Beclin1 are reduced in AD patient brains, and its depletion in APP transgenic mice decreases autophagy induction and promotes Aβ plaque deposition (Pickford et al., 2008). Vice-versa, Beclin1 mutation inducing constitutively active autophagy decreases Aβ plaque burden and memory deficits in AD mouse models (Rocchi et al., 2017), as does the enhancement of lysosomal activity (Yang et al., 2011; Xiao et al., 2015). Recent evidence indicates that AP-2 regulates the neuronal intracellular trafficking of the β-secretase BACE1 and promotes its delivery to lysosomes for degradation: conditional AP-2 knock-out mice show an accumulation of BACE1 in autophagosomes and endosomes and an increase in Aβ production (Bera et al., 2020). Accordingly, in APP transgenic mice, the enhancement of BACE1 retrograde transport decreases Aβ production and deposition, ameliorates synapse loss, and mitigates cognitive impairment (Ye et al., 2017). Interestingly, AP-2 levels are reduced in iPSC-derived neurons from AD patients (Bera et al., 2020).

Impaired mitophagy is also implicated in AD and results in the accumulation of damaged mitochondria in the hippocampus of AD patients. Mitophagy enhancement ameliorates memory impairment in AD animal models and, interestingly, reduces Aβ deposition and tau hyperphosphorylation (Fang et al., 2019).

Hence, growing evidence supports a role for autophagy and lysosomal enhancement as a therapeutic strategy in AD. Autophagy boosting through intracerebroventricular injection of the disaccharide trehalose (Du et al., 2013) or administration of mTOR-dependent autophagy activators, such as arctigenin, temsirolimus and rapamycin, showed positive effects in AD mouse models, by limiting Aβ production and plaque load and ameliorating cognitive deficits (Spilman et al., 2010; Zhu et al., 2013; Jiang et al., 2014). It cannot be underestimated, however, that this strategy may be beneficial only early in the disease (Majumder et al., 2011), when neuronal loss has not yet occurred. In addition, since in AD brains autophagosome clearance seems to be impaired, it is also possible that strong autophagy induction could exacerbate the already existing neuronal defects. In this sense, lysosomal potentiation (e.g., through viral-mediated overexpression of the lysosomal master regulator TFEB) might be advantageous (Xiao et al., 2015). Concerning the translation of these results to humans, a phase I clinical trial based on oral administration of rapamycin to patients with early onset AD is about to start (NCT04200911).

### Parkinson’s Disease

Parkinson’s disease is characterized by dopaminergic neuronal loss in the substantia nigra and formation of intracellular α-synuclein inclusions, known as Lewy bodies. α-synuclein is itself a substrate of autophagy, but compelling evidence has shown that α-synuclein aggregates directly alter the autophagic-lysosomal pathway. Reduced ATG7 and increased mTOR levels in patients’ brains and α-synuclein transgenic mice suggest a deficiency in autophagy initiation in neurons bearing α-synuclein inclusions (Crews et al., 2010). Consistent with that, α-synuclein overexpression in cell lines inhibits autophagosome biogenesis (Winslow et al., 2010). α-synuclein aggregates also disrupt the retrograde transport of AVs, thus impairing autophagosome maturation and fusion with lysosomes (Tanik et al., 2013; Volpicelli-Daley et al., 2014).

Genetic studies have identified several genes involved in autophagy and lysosomal biology as a risk factor for PD, including those encoding the lysosomal enzymes cathepsin B and glucocerebrosidase, the vacuolar ATPase subunit ATP6V0A1, and the lysosomal K^+^ channel TMEM175 (Chang et al., 2017). Moreover, many genes associated with familial PD, such as those encoding synaptojanin 1, LRRK2, the lysosomal ATPase ATP13A2 (or PARK9), PINK1 and Parkin, are involved in the autophagic and lysosomal pathways (Hunn et al., 2015; Beilina and Cookson, 2016). In particular, LRRK2 localizes to AVs and its knock-down promotes autophagy in cell lines (Alegre-Abarrategui et al., 2009), but the exact mechanism of autophagy regulation by LRRK2 remains unclear. Idiopathic PD brains and transgenic mice carrying LRRK2 mutations exhibit autophagic, endolysosomal and mitochondrial abnormalities (Ramonet et al., 2011; Rocha et al., 2020). Analogously, PD-related mutations in ATP13A2 cause an impairment of lysosomal function, thus promoting AV and α-synuclein accumulation (Dehay et al., 2012; Usenovic et al., 2012). Interestingly, ATP13A2 depletion causes the reduction of another PD-associated protein, synaptotagmin-11, and this in turn seems to be responsible for the impairment of lysosomal function and autophagosome clearance. Indeed, the overexpression of synaptotagmin-11 in ATP13A2 knocked-down cells rescued the autophagic flux, lysosomal function and α-synuclein clearance, indicating that the two PD-related proteins act in the same pathway (Bento et al., 2016).

Several strategies to boost autophagy and lysosomal function have been tested in PD models. As previously mentioned, a master regulator of the autophagy-lysosomal pathway is the transcription factor TFEB, whose nuclear localization is decreased in PD postmortem midbrains. Viral-mediated TFEB overexpression in a rat model of α-synuclein toxicity stimulated autophagy and lysosomal function, reduced α-synuclein oligomers and conferred neuroprotection to nigral dopaminergic neurons (Decressac et al., 2013). Similar results were obtained upon Beclin-1 gene transfer (Spencer et al., 2009; Decressac et al., 2013). Treatment of different animal models with autophagy-enhancing agents such as trehalose, metformin, rapamycin and temsirolimus efficiently attenuated motor dysfunctions, improved the survival of dopaminergic neurons and reduced α-synuclein aggregates (Crews et al., 2010; He et al., 2016; Lu et al., 2016; Siracusa et al., 2018). In addition, the fact that dopaminergic neurons seem to be particularly vulnerable to mitochondrial dysfunction and that two genes associated with familial PD, Parkin and PINK1, are involved in mitophagy suggests that the development of specific strategies to stimulate mitophagy may provide new therapeutic opportunities for PD (Bingol and Sheng, 2016).

### Huntington’s Disease

Huntington’s disease is a polyglutamine (polyQ) disorder caused by a CAG expansion in the Huntingtin gene that renders the mutant protein prone to aggregation (Arrasate and Finkbeiner, 2012). Extensive evidence for autophagy dysfunction has been reported in HD. The expression of several autophagic markers is dysregulated in HD human brains (Martin et al., 2015), and the V471A polymorphism in ATG7, involved in autophagosome biogenesis, is associated with an earlier onset of HD in the Italian population (Metzger et al., 2013). Wild-type Htt (wtHtt) itself exerts several important functions in neuronal autophagy, as it interacts with various autophagic components (Ochaba et al., 2014). Htt is required for stress-induced selective autophagy, such as mitophagy and aggrephagy, as it facilitates cargo recognition and sequestration by interacting with the autophagy receptor p62 (Rui et al., 2015). Accordingly, the presence of empty autophagosomes and the accumulation of dysfunctional mitochondria has been reported in cellular and animal HD models (Martinez-Vicente et al., 2010; Franco-Iborra et al., 2020). Additionally, wtHtt interacts with the autophagy-activating kinase ULK1 and promotes its activation by interfering with the inhibitory binding of mTOR to ULK1 itself, thus regulating selective autophagy initiation (Rui et al., 2015). Furthermore, the protein is involved in the axonal retrograde transport of autophagosomes, which is disrupted in Htt-depleted neurons or neurons expressing the polyQ mHtt, thus leading to an accumulation of autophagosomes and to an inefficient cargo degradation (Wong and Holzbaur, 2014a).

From a therapeutic perspective, the modulation of autophagy showed promising effects in HD models. Autophagy stimulation with rapamycin decreased polyQ aggregates and attenuated HD in different models (Ravikumar et al., 2004; Sarkar et al., 2008). Trehalose also proved beneficial in HD mouse models (Tanaka et al., 2004), but the contribution of autophagy induction was not addressed in this study. Targeted therapies can also be envisaged and may represent a valid option for HD. In this sense, a recent elegant study identified a mHtt-LC3 linker compound that interacts with the expanded polyQ-tract of mHtt and promotes its selective autophagic degradation, while preserving wtHtt. This compound proved capable of rescuing relevant HD phenotypes in fly and mouse models of the disease (Li et al., 2019).

### Amyotrophic Lateral Sclerosis

Amyotrophic lateral sclerosis is characterized by the progressive loss of motoneurons and by the accumulation of aggregated proteins such as TDP-43 or mutant SOD1 and FUS (Taylor et al., 2016). Familial and sporadic cases have been associated with mutations in genes encoding autophagy cargo receptors, such as p62/SQSTM1 and optineurin (Maruyama et al., 2010; Fecto et al., 2011; Rubino et al., 2012; Yilmaz et al., 2020). Various mutations in p62 are located in the ubiquitin-associated domain or in the LC3-interacting motif, pointing to impaired cargo recruitment to autophagosomes in ALS (Teyssou et al., 2013; Goode et al., 2016). Similarly, ALS-linked mutations in optineurin interfere with the clearance of protein inclusions and of damaged mitochondria in cell lines and neurons (Wong and Holzbaur, 2014b; Shen et al., 2015; Evans and Holzbaur, 2020). Along the same line, ALS mutations in the TBK1 kinase have also been identified (Cirulli et al., 2015; Freischmidt et al., 2015; Liu et al., 2020). TBK1 phosphorylates p62 and optineurin, thus increasing their ability to bind ubiquitinated proteins and enhancing the autophagic clearance of target proteins and mitochondria (Pilli et al., 2012; Matsumoto et al., 2015; Richter et al., 2016). TBK1 variants impact on these functions (Freischmidt et al., 2015; Foster et al., 2020). Additional genes mutated in ALS and involved in the autophagic pathway include Ubiquilin-2 and C9Orf72, the latter being affected by an intronic hexanucleotide repeat expansion that is the most common genetic abnormality in familial ALS (Deng et al., 2011; Renton et al., 2011). Ubiquilin-2 binds ubiquitinated proteins and is implicated in aggregate clearance through the proteasomal system (Hjerpe et al., 2016), but it also interacts with LC3 and with the ATP6V1G1 subunit of the vacuolar ATPase. Indeed, its depletion or mutation impair autophagosome maturation and acidification (N’Diaye et al., 2009; Wu et al., 2020). C9Orf72, in a complex with SMCR8 and WDR41, participates in autophagy initiation by activating Rab8a and Rab39b and regulating ULK1, and its loss in neurons leads to the accumulation of protein aggregates (Sellier et al., 2016; Webster et al., 2016; Yang et al., 2016). Puzzling results have shown, however, that C9Orf72 depletion reduces mTORC1 activity and increases lysosomal biogenesis at the transcriptional level, thus pointing to a negative role of C9Orf72 in the autophagic pathway (Ugolino et al., 2016; Wang et al., 2020). Lysosomal deficits, autophagosome accumulation, mitochondrial pathology and impaired retrograde transport of late endosomes have also been reported in the hSOD1^*G*93*A*^ transgenic mouse model. Interestingly, boosting retrograde transport through the overexpression of the dynein adaptor snapin rescued these defects, improved motoneuron survival and reduced disease progression in this model (Xie et al., 2015).

Despite strong genetic evidence pointing to defective autophagy as a central mechanism in ALS, conflicting results have been obtained with the pharmacological modulation of this pathway in ALS models. Autophagy induction with rapamycin or other autophagy enhancers ameliorates TDP-43 clearance and neuronal survival in cellular and animal models, and significantly improves motor and cognitive dysfunctions in TDP-43 transgenic mice (Wang et al., 2012; Barmada et al., 2014). Conversely, rapamycin showed detrimental effects in hSOD1^*G*93*A*^ mice (Zhang et al., 2011), while autophagy dampening through the heterozygous deletion of Beclin1 increases the life span of the SOD1^*G*86*R*^ model (Nassif et al., 2014). mTOR-independent induction of autophagy with trehalose, instead, reduced protein aggregation, enhanced motoneuron survival, increased life span, and reduced disease progression in mutant SOD1 models (Castillo et al., 2013; Zhang et al., 2014).

## Conclusion

In recent years there has been increasing demonstration of autophagy/endolysosomal dysfunction as a pathogenic mechanism shared by multiple neurodegenerative diseases, likely contributing to synaptic dysfunction and ultimately neuronal death. Interestingly, the selective vulnerability of different neuronal populations has been suggested to rely on intrinsic differences in the efficiency of these proteostasis systems (Diedrich et al., 2011; Tsvetkov et al., 2013). Still, in most cases we lack a thorough description of the molecular mechanisms by which specific disease-associated variants in autophagy genes may cause synaptic defects and, importantly, we lack a precise definition of the extent to which specific synaptic dysfunctions can be reverted by restoring autophagic function. This knowledge will probably be instrumental for the development of new and more specific drugs/approaches: autophagy induction alone may not be sufficient, or even detrimental in some cases, and the modulation of selective autophagic pathways may be advantageous compared to a perturbation of general autophagy.

## Author Contributions

SG, FG, and MR wrote the first draft of the manuscript and prepared the figure. All authors participated to literature search, revised the text, and approved the final version of the manuscript.

## Conflict of Interest

The authors declare that the research was conducted in the absence of any commercial or financial relationships that could be construed as a potential conflict of interest. The reviewer FC declared a past co-authorship with one of the authors SG to the handling editor.
